# Four cases of gastric adenocarcinoma and proximal polyposis of the stomach treated by robotic total gastrectomy

**DOI:** 10.1186/s40792-022-01425-6

**Published:** 2022-04-18

**Authors:** Yosuke Iwakawa, Kozo Yoshikawa, Koichi Okamoto, Tetsuji Takayama, Takuya Tokunaga, Toshihiro Nakao, Masaaki Nishi, Chie Takasu, Hideya Kashihara, Yuma Wada, Toshiaki Yoshimoto, Shoko Yamashita, Mitsuo Shimada

**Affiliations:** 1 The Department of Surgery, The University of Tokushima, 3-18-15 Kuramoto-cho, Tokushima, Japan; 2 The Department of Gastroenterology and Oncology, Institute of Biomedical Sciences, Tokushima University Graduate School, Kuramoto-cho, Tokushima, 770-8503 Japan

**Keywords:** GAPPS, Robotic total gastrectomy, Mutation of APC exon 1B

## Abstract

**Background:**

Gastric adenocarcinoma and proximal polyposis of the stomach (GAPPS) is a rare disease and characterized by a unique point mutation in the promoter 1B region of the adenomatous polyposis coli (APC) gene. There are two aims in surgery for GAPPS; the first is prophylactic gastrectomy, and the second is excising concurrent cancer. We performed robotic total gastrectomy (RTG) for four cases of GAPPS.

**Case presentation:**

Case 1 was a woman in her 40 s whose sister had died from gastric cancer. Mutational analysis revealed mutation of APC exon 1B. We performed prophylactic gastrectomy. Case 2 was a woman in her 30 s who had a mutation of APC exon 1B, and preoperative biopsy revealed suspected adenocarcinoma. Case 3 was a woman in her 40 s who was diagnosed with gastric cancer with multiple polyps in the stomach and a mutation of APC exon 1B. Case 4 was a woman in her 20 s in whom biopsy revealed low-grade dysplasia of a raised lesion. She had a mutation in APC exon 1B. We performed RTG with D1 + lymphadenectomy in all patients, and there were no intraoperative complications.

**Conclusions:**

Patients with GAPPS are mainly followed regularly with repeat biopsy, and tumors are detected in an early stage. As the safety of robotic surgery for the early gastric cancer is reported, RTG is an option for these patients. This is the first report of RTG for GAPPS patients.

## Background

Gastric adenocarcinoma and proximal polyposis of the stomach (GAPPS) is a rare disease [1]. GAPPS is diagnosed in accordance with the following clinical and pathologic criteria: (1) gastric polyps restricted to the body and fundus with no evidence of colorectal or duodenal polyposis; (2) > 100 polyps carpeting the proximal stomach in the index case or > 30 polyps in a first-degree relative; (3) predominantly fundic gland polyps (FGPs), some having regions of dysplasia, or a family member with either dysplastic FGPs or gastric adenocarcinoma; and (4) an autosomal dominant pattern of inheritance. Other causes of FGPs, such as a proton pump inhibitor (PPI) effect and heritable gastric polyposis syndromes, must be excluded [2]. Genetically distinct from classic familial adenomatous polyposis (FAP) and attenuated FAP, GAPPS is characterized by a unique point mutation in the promoter 1B region of the adenomatous polyposis coli (APC) gene [3].

Patients with GAPPS are sometimes followed by endoscopic examination, and suspected cancer lesions are evaluated. As a result, patients requiring surgery undergo prophylactic gastrectomy or gastrectomy for early gastric cancer lesions. Recently, robotic gastrectomy has been performed for gastric cancer, and in early gastric cancer, the method’s safety and usefulness have been proven [4]. Therefore, robotic gastrectomy may be beneficial for patients with GAPPS. We report four cases of robotic total gastrectomy (RTG) for GAPPS.

## Case presentation

### Case 1

**Fig. 1:**
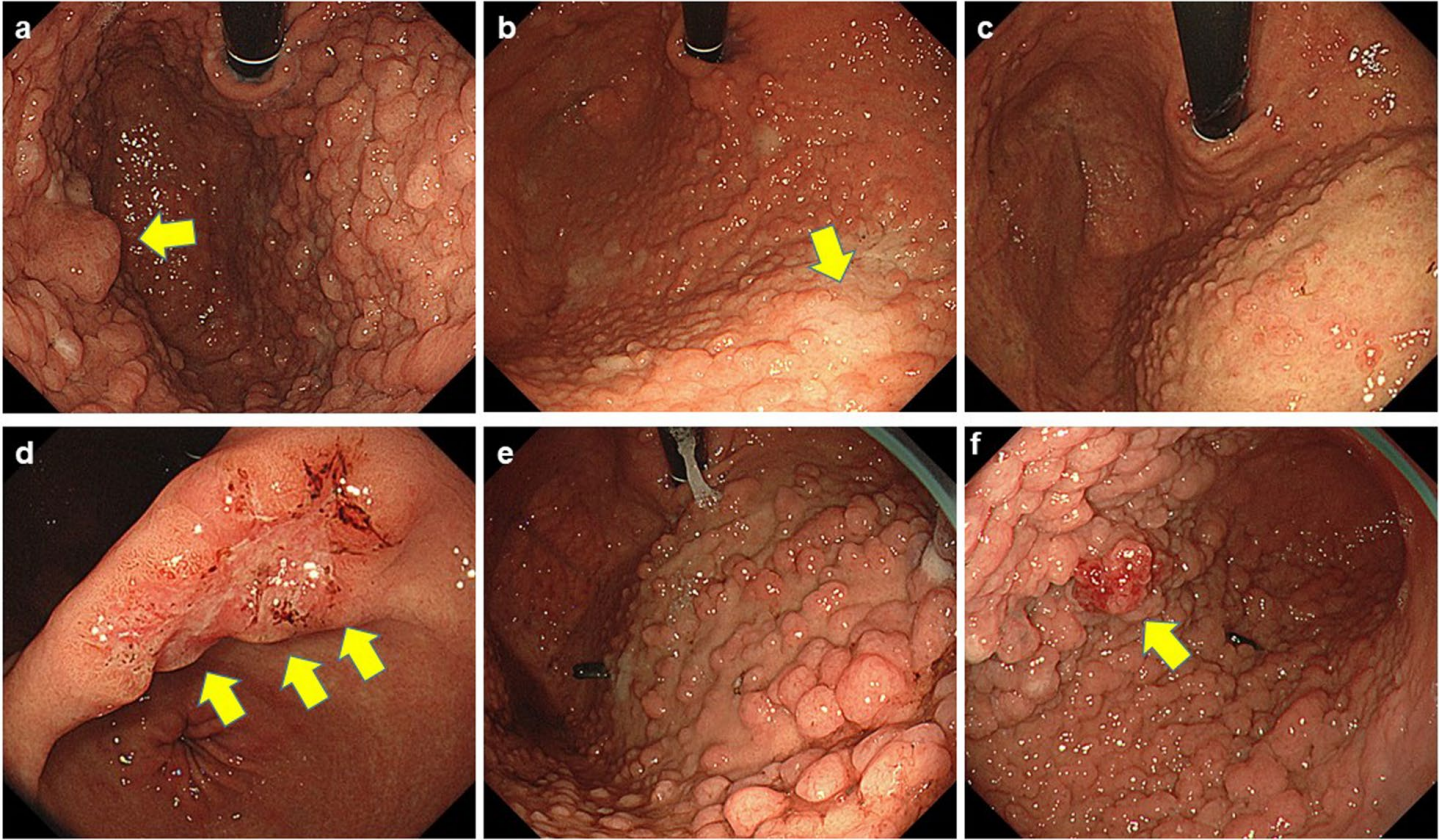
Esophagogastroduodenoscopy. **a** Case 1; **b** Case 2, cancer-suspected lesion (arrow); **c** Case 3; **d** adenocarcinoma in Case 3 (arrow); **e** Case 4; **f** raised lesions in the gastric body in Case 4 (arrow)

**Fig. 2:**
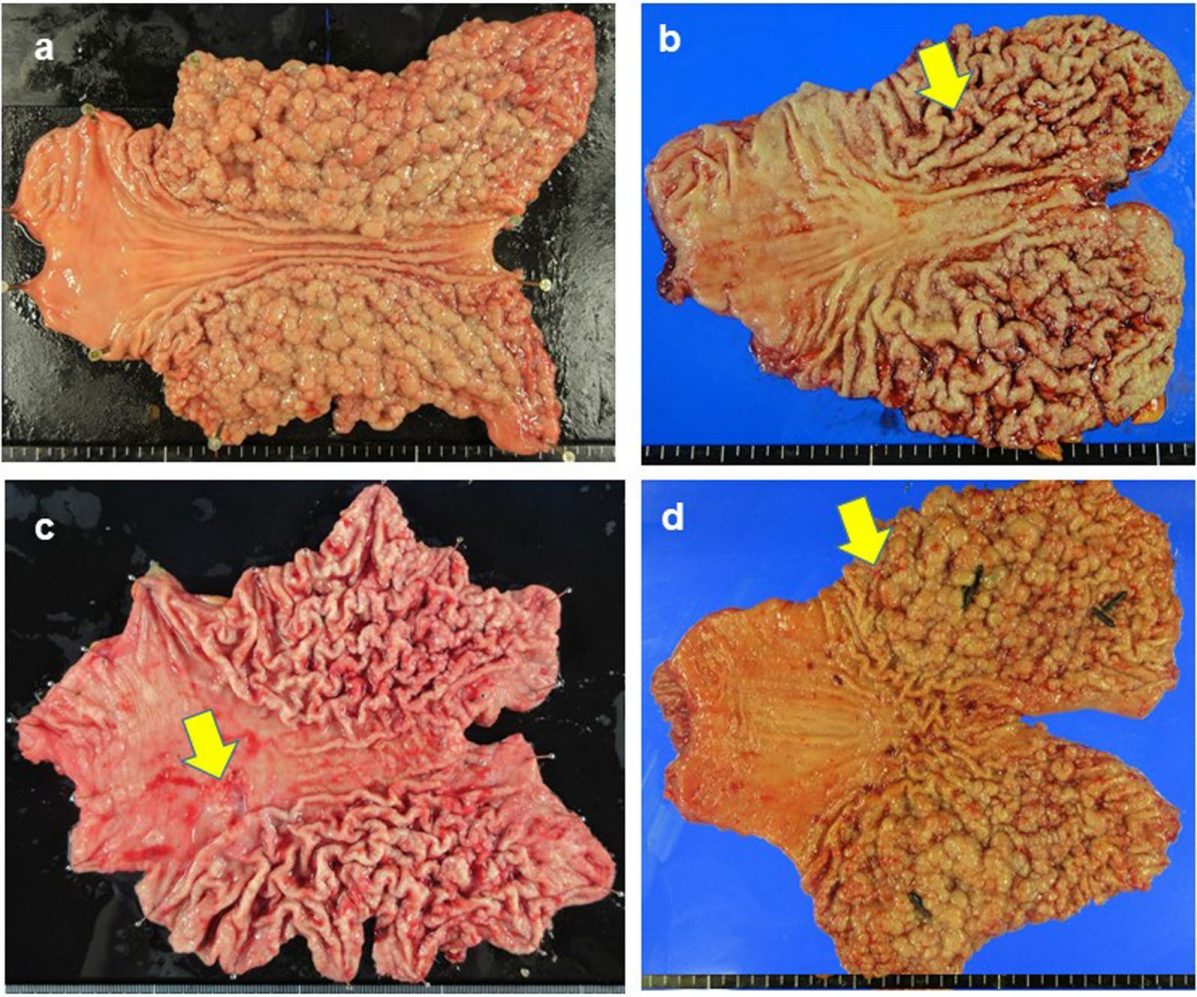
Surgical specimen. **a** Case 1; **b** Case 2, cancer-suspected lesion (arrow); **c** Case 3, cancer lesion (arrow); **d** Case 4, cancer-suspected lesion (arrow)

A woman in her 40 s presented to our hospital because of abnormal shadows detected in an upper gastrointestinal series. Her medical history included depression and PPI administration. Her sister had died from gastric cancer, and FGPs were identified. Her father had adenomatosis polyposis, and her cousins had GAPPS. Laboratory test results were as follows: carcinoembryonic antigen (CEA): 1.2 ng/ml, carbohydrate antigen 19-9 (CA19-9): 1 U/ml, CA125: 22 U/ml, and CA72-4: 2.6 U/ml; *Helicobacter pylori* (H. pylori) testing was negative. Esophagogastroduodenoscopy (EGD) showed multiple FGPs in the fornix and fundus (Fig. 1a) but no abnormal findings in the antrum and duodenum. Pathologically, biopsies indicated FGPs. Immunostaining was positive for MUC5, MUC6, MUC2, p53, and CDX2. Total colonoscopy (TCS) and computed tomography (CT) showed no abnormal findings. There was minimal ascites but no swollen lymph nodes. Considering her family history, FGPs revealed by EGD, and no apparent polyposis in the colon or duodenum. We performed mutational analysis, which revealed a mutation of APC exon 1B; thus, she was diagnosed with GAPPS. We performed prophylactic RTG with D1 + lymphadenectomy (Fig. 2a). The operation time was 337 min, and blood loss was 45 ml. Histopathological examination of the surgical specimens revealed carpet-like polyposis as a common finding of GAPPS. The polyposis was FGP and was considered adenoma. Immunostaining was positive for MUC6, MUC5AC, p53, and Ki-67 and negative for cluster of differentiation (CD)10, CDX2, and MUC2. She had no recurrence 3 years after surgery.

### Case 2

A woman in her 30 s presented to our hospital for burping. She had no remarkable medical history, and PPI administration was ineffective. Her grandfather had lung cancer, and her grandmother had undergone gastrectomy; however, it was unclear whether her grandmother had gastric cancer. Laboratory test results were as follows: CEA: 0.4 ng/ml, CA19-9: 8 U/ml, CA125: 13 U/ml, and CA72-4: 1.9 U/ml; *H. pylori* testing was negative. EGD showed multiple FGPs (Fig. 1b) and type 0–I lesions that were suspected gastric cancer (Fig. 1b, arrow). There were no abnormal findings in the duodenum. Pathologically, biopsies indicated FGPs. Type 0–I lesions indicated foveolar-type dysplasia/adenoma or adenocarcinoma with low-grade atypia. Immunostaining was positive for β-catenin, MUC5AC, MUC6, p53, and Ki-67. TCS and CT showed no abnormal findings. There was minimal ascites, but no swollen lymph nodes. Considering her family history and the EGD findings, we suspected GAPPS. We performed mutational analysis of the APC gene, which revealed mutation of APC exon 1B. Thus, she was diagnosed with GAPPS. We performed RTG with D1 + lymphadenectomy (Fig. 2b) owing to the diagnosis of adenocarcinoma suspected by biopsy. The operation time was 285 min, and blood loss was 13 ml. Histopathologically, the surgical specimens revealed FGPs with dysplasia in the fundus. Immunostaining was positive for MUC5AC, p53, and Ki-67. Staining for MUC6, CDX2, and MUC2 was negative; therefore, we diagnosed GAPPS. She had no recurrence 1 year after surgery.

### Case 3

A woman in her 40 s presented to our hospital for early gastric cancer with suspected GAPPS. She had no remarkable medical history, but had undergone eradication therapy for *H. pylori* 6 years earlier. Her father had been diagnosed with early gastric cancer. Laboratory test results were as follows: CEA: 1.3 ng/ml, CA19-9: 10 U/ml, CA125: 18 U/ml, and CA72-4: 10.5 U/ml. EGD showed multiple FGPs in the upper stomach (Fig. 1c) and early gastric cancer in the gastric angle (Fig. 1d). There were no abnormal findings in the duodenum. Biopsy revealed tub1 in the gastric cancer lesion. TCS revealed two polyps, and CT identified no other tumors and no swollen lymph nodes. We suspected GAPPS and performed mutational analysis of APC exon 1B, which revealed a mutation. Thus, she was diagnosed with GAPPS with early gastric cancer. We performed RTG with D1 + lymphadenectomy (Fig. 2c). The operation time was 281 min, and blood loss was 10 ml. Histopathologically, the surgical specimens revealed early gastric cancer (T1b2) with no lymph node metastasis. She had no recurrence 2 years after surgery.

### Case 4

A woman in her 20 s presented to our hospital for screening because her aunt had been diagnosed with GAPPS and gastric cancer. She had no remarkable medical history, and PPI administration had no effect. Her grandmother had gastric cancer, and her father and aunt had GAPPS. Laboratory test results were as follows: CEA: 0.5 ng/ml, CA19-9: 17 U/ml, CA125: 28 U/ml, and CA72-4: 7.8 U/ml; *H. pylori* testing was negative. EGD showed multiple FGPs (Fig. 1e) and raised lesions in the gastric body (Fig. 1f). There were no abnormal findings in the duodenum. We performed endoscopic mucosal resection for the raised lesions. Pathologically, the biopsies indicated FGP in the polyposis lesions and low-grade dysplasia in the raised lesions. Immunostaining was positive for MUC5AC, MUC6, and Ki-67. Scanning for MUC2 and CDX2 was negative. TCS showed no abnormal findings, and CT did not reveal other tumors. There was minimal ascites, but no swollen lymph nodes. Considering her family history and the EGD findings, we suspected GAPPS. We performed mutational analysis of APC exon 1B, which revealed a mutation. Thus, she was diagnosed with GAPPS, and we performed RTG with D1 + lymphadenectomy (Fig. 2d). The operation time was 288 min, and blood loss was 35 ml. Histopathologically, the surgical specimens revealed hyperplasia of gastric fundic gland tissue and low-grade dysplasia. Immunostaining was positive for MUC5AC, MUC6, p53, and Ki-67. MUC2 and CDX2 immunostaining was negative. She had no recurrence 2 months after surgery.

## Discussion

GAPPS is an autosomal dominant syndrome characterized by FGPs localized in the gastric body and fundus. GAPPS was first reported by Worthly et al. in 2012 [1]. Since then, there have been an increasing number of reports on GAPPS [5–9]; however, the clinical course, malignant transformation, and prognosis of GAPPS remain unclear. The prognosis of GAPPS depends on the malignancy of the FGPs. However, the clinical management of GAPPS is difficult because it is difficult to assess the exact status of the FGPs and to predict their malignant transformation. EGD is useful for early detection of GAPPS, and regular biopsy is necessary to predict malignant transformation. However, cases of rapid progression to gastric adenocarcinoma and metastasis despite frequent EGD have been reported. *H. pylori* infection suppresses FGPs, and patients with FAP who have FGPs have a lower H. pylori infection rate [1, 3, 7]. Furthermore, H. pylori is closely associated with GAPPS, although many patients with GAPPS have negative immunoglobulin G test results for H. pylori. However, the association between H. pylori infection and gastric cancer development is still unclear, and the usefulness of H. pylori eradication therapy for GAPPS is unknown [10, 11].

Histopathologically, FGP is a characteristic feature of GAPPS and is included in the diagnostic criteria. Hyperproliferative aberrant pits, which are polyp-like structures caused by irregular growth of proper gastric glands in diluted glandular fossa epithelialization, are also characteristic of GAPPS [7]. Immunohistologically, MUC5A and MUC6 are positive, while MUC2 is negative, and CDX2 and p53 are sporadically positive, indicating gastric-type adenocarcinoma [12]. Notably, lesions that are positive for MUC5A but negative for MUC2/MUC6 with elevated Ki-67 and strong proliferative potential have been reported. As the causative gene for GAPPS, a point mutation in APC promotor 1B has been reported, which is thought to contribute to tumorigenesis via β-catenin and Wnt signaling [13].

There are two aims in surgery for GAPPS: the first is prophylactic gastrectomy, and the second is excising concurrent cancer. In our four cases, one patient underwent prophylactic gastrectomy, one had gastric cancer, and the remaining two had suspected gastric cancer.

Regarding prophylactic TG for GAPPS, patients who fulfill the diagnostic criteria for GAPPS and those with FGPs progressing to dysplasia should undergo prophylactic TG [14]. Recently, this has been performed in Asian countries [5]. Prophylactic gastrectomy is associated with the risk of postoperative complications, such as infections, dumping syndrome, and weight loss. Furthermore, anastomotic strictures, bile reflux, and iron deficiency have also been reported as postsurgical complications of prophylactic gastrectomy [15]. Generally, prophylactic gastrectomy is performed for patients who are younger and healthier than those who undergo curative gastrectomy for gastric cancer. However, the postoperative complications after prophylactic gastrectomy are the same as those associated with curative gastrectomy [16]. Prophylactic TG for GAPPS remains controversial because GAPPS may progress rapidly even with regular surveillance. The ideal timing for prophylactic gastrectomy is also important. Prophylactic gastrectomy should be considered when GAPPS is diagnosed, but the timing of dysplasia and adenocarcinoma development varies widely. Therefore, the optimal timing of prophylactic gastrectomy is still controversial.

Regarding the treatment of concurrent suspected or confirmed gastric cancer with GAPPS, generally, these patients are mainly followed regularly with repeat biopsy. Therefore, even if adenocarcinoma is diagnosed, tumors are detected in an early stage.

In GAPPS patients, polyps are located in the upper stomach; therefore, TG is necessary. Although laparoscopic TG has recently been performed safely, RTG is challenging. Robotic surgery has several technical advantages compared with laparoscopic instruments, namely greater precision of the operator’s movements, tremor filtration, and improved ergonomics. These technical benefits are considered advantages of RTG over laparoscopic TG. Retrospective study has showed the lower complication rate in early gastric cancer patients with RTG, especially there was no leakage of anastomotic site. This study indicated the merit of the RTG was minimal damage of pancreas and lower incidence of pancreatic fistula [17]. In GAPPS patients, polyps are not located in the esophagus; therefore, TG is relatively easy to perform compared with esophagogastric junction cancer, making robotic gastrectomy useful. Furthermore, for relatively young patients undergoing surgery, cosmesis is more of a concern; therefore, minimally invasive surgery is useful.

**Fig. 3:**
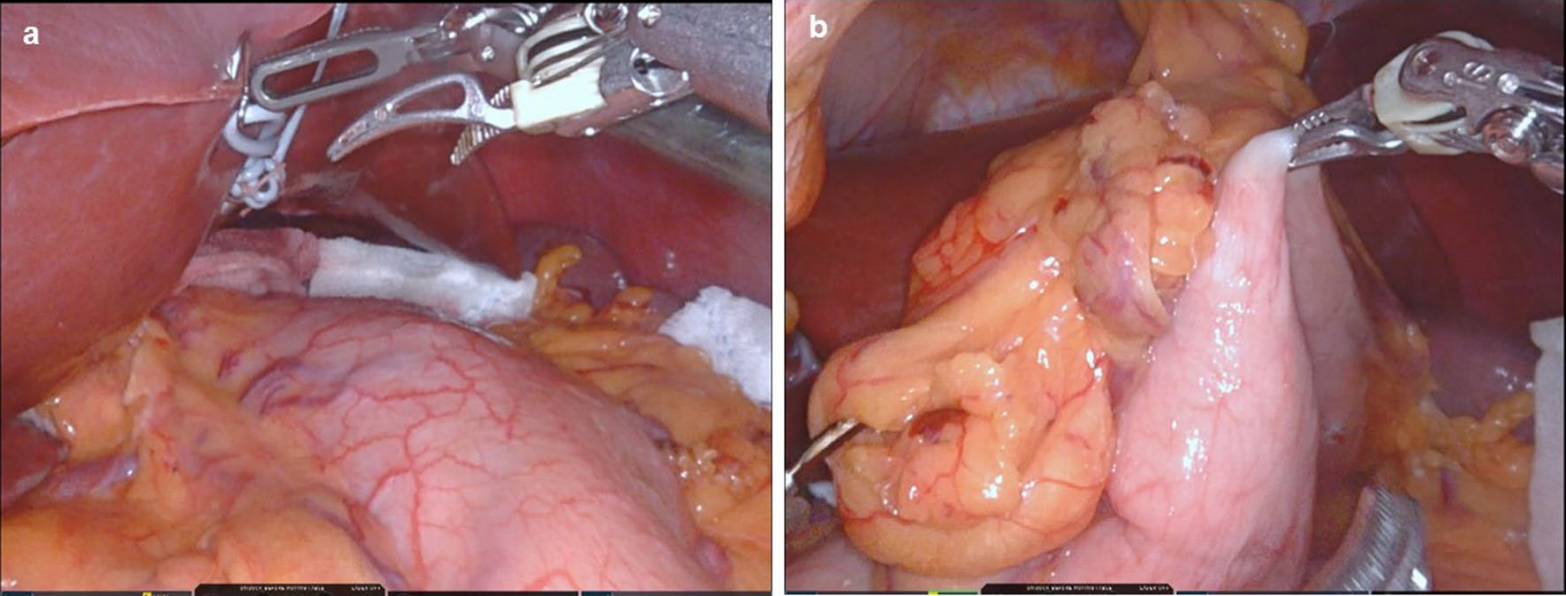
Intraoperative finding. **a** Appearance of stomach wall, **b** stomach wall grasped by robotic forceps

In gastrectomy in GAPPS patients, although there are numerous polyps in the stomach, the serosa remains intact, and there is no stiffness of the stomach wall (Fig. 3a). As a result, it is easy to grasp the stomach wall (Fig. 3b). We encountered no stomach wall stiffness in our operations. This information is useful for the surgeons because GAPPS is a rare disease, few surgeons have experience with these patients.

To our knowledge, there are no reports of GAPPS patients undergoing robotic gastrectomy.

## Conclusions

We reported four cases of GAPPS patients who underwent RTG, which is an option for these patients.

## Data Availability

Not applicable.

## Abbreviations

FGP: Fundic gland polyps
*H. pylori*: *Helicobacter pylori*
FAP: Familial adenomatous polyposis
APC: Adenomatous polyposis coli
GAPPS: Gastric adenocarcinoma and proximal polyposis of the stomach
RTG: Robotic total gastrectomy
PPI: Proton pump inhibitor
CEA: Carcino-embryonic antigen
CA19-9: Carbohydrate antigen19-9
CA125: Carbohydrate antigen125
CA72-4: Carbohydrate antigen72-4
EGD: Esophagogastroduodenoscopy
TCS: Total colonoscopy
CT: Computed tomography
